# Effective Control of Pancreatitis-Related Gastric Varices Through Splenic Artery Embolization: A Case Report

**DOI:** 10.7759/cureus.69519

**Published:** 2024-09-16

**Authors:** Jerry Cruz Rodriguez, Isabel Castellanos-Castillo, Carlos J Pérez Refojos, José C Jiménez García

**Affiliations:** 1 Medicine, Universidad Central del Caribe, San Juan, PRI; 2 Medicine, Universidad Central Del Caribe, San Juan, PRI; 3 Diagnostic Radiology, University of Puerto Rico, Medical Sciences Campus, San Juan, PRI; 4 Gastroenterology, Auxilio Mutuo Hospital, San Juan, PRI

**Keywords:** bleeding gastric varices, chronic pancreatitis (cp), gastric varices, isolated gastric varices, splenic artery embolization, splenic vein obstruction, type 1 isolated gastric varices, type 2 isolated gastric varices

## Abstract

The development of isolated gastric varices (IGVs) is a rare manifestation in the setting of chronic pancreatitis (CP). However, due to the proximity of the pancreas to the splenic vein, splenic vein thrombosis (SVT) secondary to compression from CP can lead to the development of IGVs. We report a case of a 57-year-old patient with a history of CP status post Whipple procedure with upper GI bleeding from IGV formation. Initial endoscopic evaluation revealed scattered and prominent bleeding varies in the stomach with no evidence of esophageal variceal development. Subsequent CT angiography revealed SVT as the underlying cause of the variceal formation. The patient initially received medical management with IV sandostatin for bleeding control, and a splenectomy was planned as the definitive management. However, because the patient had undergone a Whipple procedure and developed fragile scattered varices, splenic artery embolization was chosen over splenectomy. Splenic artery embolization led to a successful reduction in gastric variceal size and no recurrence of bleeding. This case report presents a case of pancreatitis-induced splenic vein thrombosis (PISVT) and highlights splenic artery embolization as a viable management strategy in such cases.

## Introduction

The development of gastric varices signifies a serious and potentially life-threatening complication. When primarily associated with portal hypertension, it is often accompanied by other variceal formations, such as esophageal varices. Gastric varices develop from an upper venous obstruction that causes an increase in the pressure of the venous system and causes backflow to the gastric veins. This is most commonly caused by cirrhosis of the liver [1]. However, due to the proximity of the pancreas to the splenic vein, inflammation of the pancreas leading to splenic vein compression poses a significant risk for the development of gastric varices in the setting of pancreatitis. Pancreatitis-induced splenic vein thrombosis (PISVT), seen in both acute and chronic pancreatitis (CP), is a rare occurrence in which compression of the splenic vein by the inflammatory process of the pancreas or thrombosis causes an upstream increase in pressure of the splenic vein and backflow engorgement of the gastric veins [2]. Following the backflow and pressure buildup generated by PISVT, splenic venous blood is redirected, leading to the development of collateral blood flow through the splenoportal or gastroepiploic systems [2]. This results in localized venous hypertension, commonly referred to as sinistral or left-sided portal hypertension [2]. Isolated gastric varices (IGVs) develop as a result of increased blood flow through these systems during left-sided portal hypertension [2].

Isolated gastric varices are categorized into two primary types based on their anatomical location in the stomach. Fundal varices, or type 1 isolated gastric varices (IGV1), are more common and carry the highest risk of bleeding. In contrast, antral varices, or type 2 isolated gastric varices (IGV2), are relatively uncommon but have the potential for hemorrhage as well [3]. In cases of PISVT with no active bleeding, expectant management and regular variceal monitoring are typically employed. However, in the presence of bleeding or symptomatic varices, splenectomy is the recommended approach for definitive resolution, following stabilization through endoscopic or medical management of the bleeding [4]. In recent years, splenic artery embolization has emerged as a more conservative option for the management of gastric varices related to PISVT. It is a minimally invasive approach that reduces venous pressure by attenuating arterial flow to the spleen [5]. This promising alternative to treating PISVT provides effective management while reducing the potential risks associated with surgical interventions such as splenectomy [5]. In this case report, we present a patient with bleeding gastric varices following a Whipple procedure for CP, managed with splenic artery embolization.

## Case presentation

**Figure 1:**
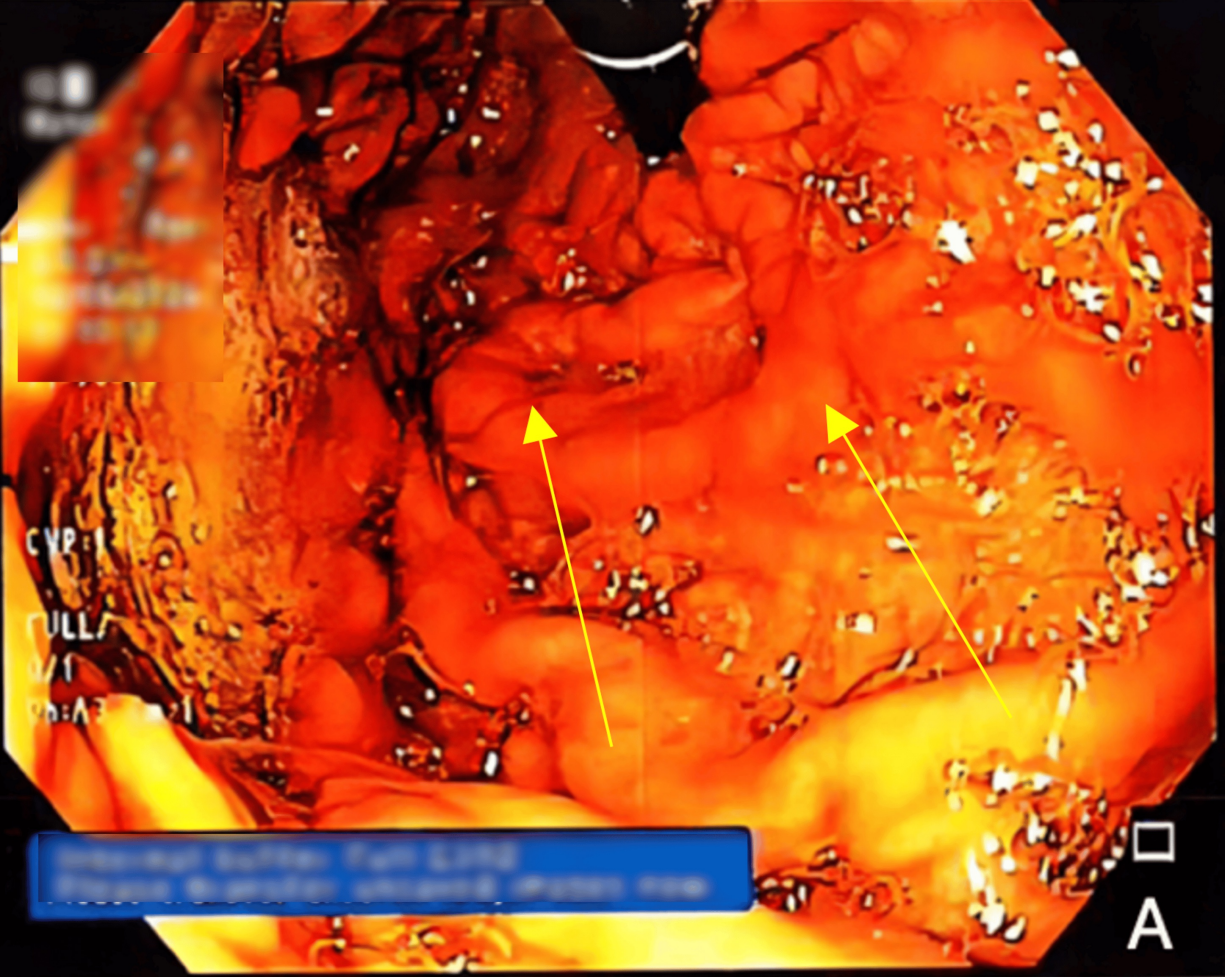
Esophagogastroduodenoscopy (EGD) findings of type 1 isolated gastric varices (IGV1) are noted.

A 57-year-old male with a medical history of HIV, hypercholesterolemia, type 2 diabetes mellitus, hypertension, and CP status post Whipple procedure two years prior presented to the emergency department with a one-week progression of dark blood in the stool, hypotension, and pain while swallowing. Physical examination was remarkable for epigastric abdominal pain for which the patient could not remember onset and conjunctival pallor. Further evaluation at the emergency department revealed a hemoglobin level of 9.8 g/dL concerning possible gastrointestinal bleeding. As the patient had no discernible abnormalities on a colonoscopy performed at the same hospital one year earlier, an esophagogastroduodenoscopy (EGD) was scheduled to assess for a possible upper GI source of the bleeding. The subsequent EGD performed during the hospital stay revealed esophageal candidiasis, which was managed with fluconazole. Notably, the EGD did not reveal any apparent esophageal varices. However, the EGD did identify prominent and nodular gastric folds along with large, congested veins, indicative of IGV1 (Figure 1).

Due to the presence of bleeding varices, the patient was transferred to the ICU, where hemoglobin levels were closely monitored throughout the hospital admission. One unit of whole blood transfusion was administered to address the declining hemoglobin levels. The patient was subsequently stabilized and was medically managed with IV sandostatin and antibiotics.

**Figure 2:**
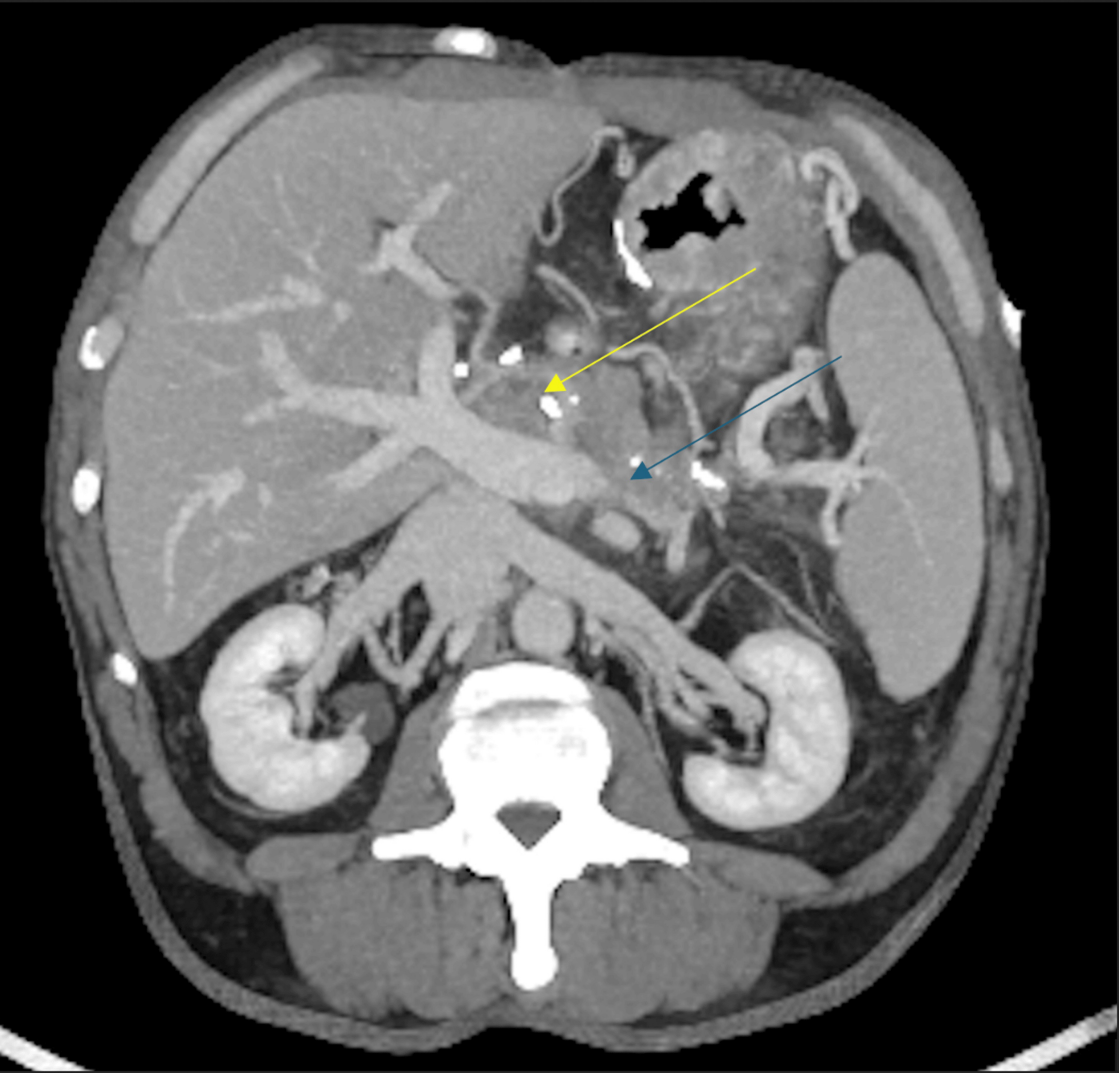
The axial, oblique view of the CT scan with IV contrast in the portal venous phase demonstrates splenic vein thrombosis and calcifications in this patient's status post Whipple procedure. The blue arrow demarcates the splenic vein thrombosis. The yellow arrow demarcates calcifications that resulted from the previous Whipple procedure.

**Figure 3:**
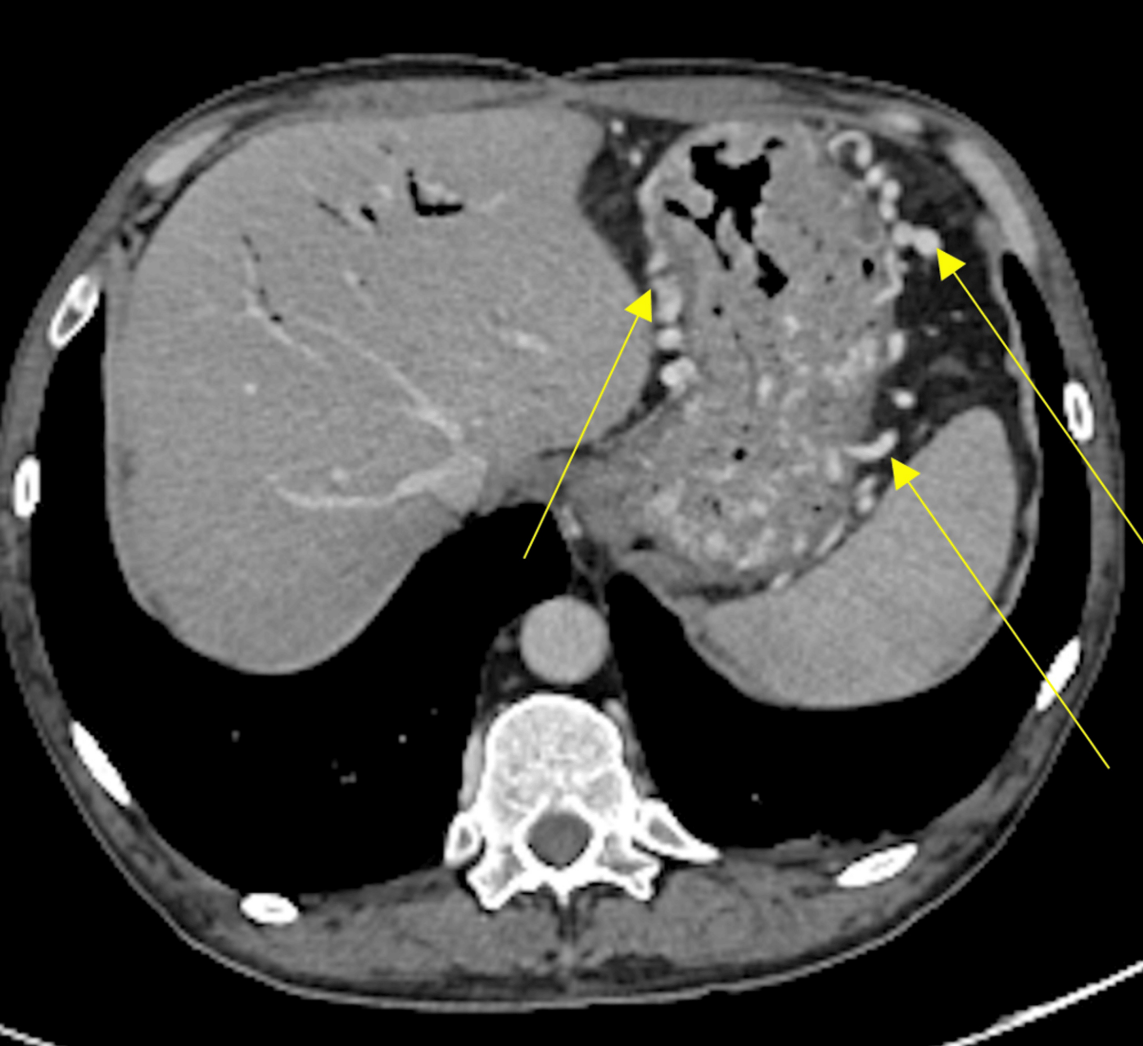
The axial view of the CT scan reveals IGV1 and IGV2 before splenic artery embolization. IGV1: isolated gastric varices type 1; IGV2: isolated gastric varices type 2

Based on the findings from the EGD and no previous history of cirrhosis or varices, a CT angiography (CTA) of the abdomen was performed to further evaluate the underlying cause of variceal development. Figure 2 illustrates a CTA showing a completely occluded splenic vein, with the thrombosis compressed by the pancreas following a Whipple procedure. No apparent splenomegaly was noted in the evaluation. Additionally, the scan identified numerous IGV1s and IGV2s secondary to chronic splenic vein thrombosis and potentially attributable to the patient's history of CP and subsequent Whipple procedure (Figure 3).

**Figure 4:**
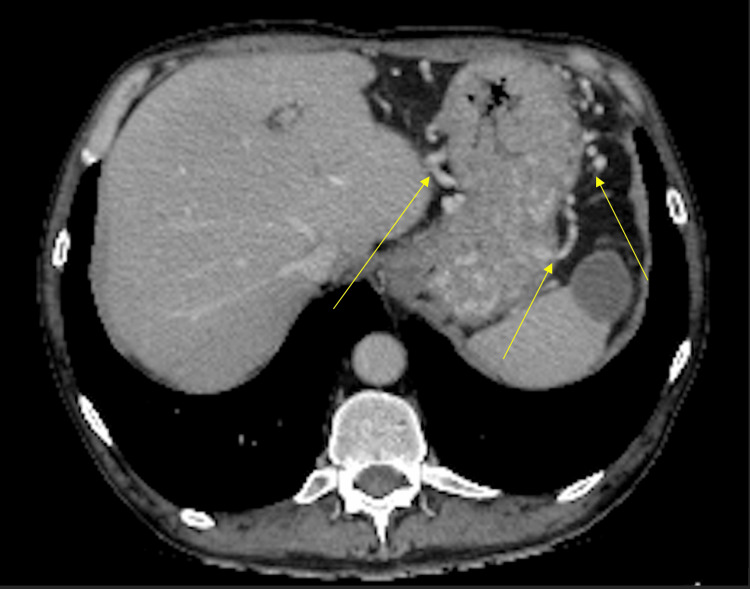
The axial view of the CT scan with IV contrast shows an interval decrease in the size of the IGV1 and IGV2 after splenic artery embolization (illustrating the same vessels as Figure 3). IGV1: isolated gastric varices type 1; IGV2: isolated gastric varices type 2 Note the splenic infarct on its anterolateral aspect.

After unrelenting symptoms and an ICU admission, a splenectomy was initially planned for definitive management. However, given the complexity associated with performing a splenectomy in a patient who had undergone a Whipple procedure and developed collateral vessels from PISVT, the multidisciplinary team, comprising gastroenterology, interventional radiology (IR), and surgery specialists, determined that partial splenic artery embolization would be a more appropriate treatment option. The IR team subsequently carried out a successful and complication-free splenic artery embolization, effectively reducing blood flow to the thrombosed splenic vein and alleviating the backflow pressure responsible for the gastric varices. Follow-up CT scans revealed interval embolization of the spleen with a subsequent decrease in the size of the dilation and the number of gastric varices (Figure 4). The patient was discharged home and advised to continue outpatient follow-up with the gastroenterology team. No further recurrence of variceal bleeding was reported by the patient on follow-up one month after the procedure. 

## Discussion

Isolated gastric varices resulting from pancreatitis represent a rare occurrence in clinical practice, constituting only 14% of all patients suffering from CP [2]. Among such cases, most present asymptomatically; however, about 12% manifest with symptoms of bleeding, anemia, hematemesis, melena, or hematochezia, thus undermining its infrequent but critical nature [2]. Gastric variceal development resulting from isolated splenic vein obstruction in PISVT, as observed in the presented case, leads to increased pressure within the short gastric and gastroepiploic veins. This phenomenon of thrombosis and backflow of venous blood through gastric veins into the portal vein is commonly referred to as left-sided portal hypertension or sinistral portal hypertension [2]. The diagnosis of left-sided portal hypertension requires a high degree of suspicion and should be considered in all patients with CP who present with upper gastrointestinal bleeding and no history of liver cirrhosis. In patients with CP, partial thrombotic occlusion of the splenic vein is significantly more common than a complete occlusion, occurring in 54% to 89% of cases [6].

An EGD is the first-line diagnostic modality for evaluating the presence of gastric varices in patients with upper GI bleeding [6]. Isolated findings of gastric varices through endoscopy should raise suspicion of possible splenic vein thrombosis (SVT) in all patients without a history of cirrhosis. Abdominal CT with contrast is the imaging modality of choice to assess for possible SVT in sinistral portal hypertension. Characteristic CT findings, such as the presence of normally enhancing portal and superior mesenteric veins, support the diagnosis of SVT [7]. Additional indicators include extensively swollen and enhancing veins in the splenic hilum, involving the short gastric, gastroepiploic, and coronary veins, or a splenic vein with a center that fails to enhance with an intravenous contrast agent [7]. However, venous phase angiography remains the gold standard confirmatory test for diagnosis and is often done on patients with obscure or acute upper GI bleeding [6-7]. Therefore, the diagnostic approach should be individualized based on the patient's medical history and symptomatology, utilizing a compilation of EGD, US, or CT scans. 

Once the diagnosis of IGV by endoscopic evaluation is established and an underlying cause is identified, treatment depends on the patient's symptomatic status. Asymptomatic patients with gastric varices induced by PISVT are generally managed with watchful waiting, beta-blocker prophylaxis, and assessment of the underlying thrombosis, while patients with minimal bleeding may be addressed with medical hemostasis using agents like octreotide and somatostatin [4, 8]. For patients with diffuse bleeding, endoscopic hemostasis with variceal ligation is typically required [8]. However, in such patients, hemorrhage from gastric varices can be life-threatening and often requires a splenectomy of splenic artery embolization as definitive management [8]. The role of prophylactic splenectomy in asymptomatic patients remains controversial [7]. Splenectomy carries several intraoperative risks, including extensive bleeding due to injury of the splenic capsule or short gastric vessels, as well as potential damage to surrounding organs such as the stomach, colon, and pancreas [9]. These complications can lead to numerous postoperative issues, including pancreatic fistulas and acute pancreatitis. Moreover, other intraoperative complications, such as splenic rupture and splenosis, can also occur [9]. Specifically, in patients with CP who have undergone a prior Whipple procedure and developed SVT, the formation of multiple collateral vessels significantly increases the risk of bleeding and other intraoperative complications during a splenectomy, requiring less invasive treatment. Any abdominal surgery is deemed high-risk for patients with severe pancreatitis. This is because of inflammation, significant adhesions, unclear anatomy, and friable tissue [10]. Splenic artery embolization may be the preferred treatment method over splenectomy for gastric variceal bleeding secondary to SVT in patients with a history of complicated or extensive abdominal surgeries. 

Splenic artery embolization is a minimally invasive endovascular procedure that is safe and effective in the management of isolated SVT from CP, as it avoids splenectomy and can effectively control gastric variceal bleeding [11]. There are no absolute contraindications for splenic artery embolization; however, it carries its own potential risk of splenic infarction, venous outflow obstruction, and post-embolization syndrome, which often resolves on its own [11]. Today, partial splenic artery embolization as the management of bleeding from large gastric varices is considered to be equally effective as splenectomy in patients with left-sided hypertension with no recurrence of bleeding [12]. Splenic arterial embolization presents itself as a promising alternative treatment for gastric varices linked to splenic vein occlusion in patients at high risk for surgery.

## Conclusions

Isolated gastric varices as a result of PISVT is a rare and life-threatening complication of CP. The formation of these varices is due to left-sided portal hypertension, leading to increased pressure and distention of gastric veins. Diagnosis of IGVs requires a high degree of clinical suspicion and a combination of endoscopic evaluation and imaging techniques. While splenectomy has been the most traditional approach for effective management as it dramatically decreases blood flow through the splenic vein, splenic artery embolization is a safe and efficient alternative in patients for whom splenectomy is contraindicated, resulting in equal outcomes.
